# A SNARC-like effect for visual speed

**DOI:** 10.3758/s13414-025-03012-x

**Published:** 2025-01-29

**Authors:** Michele Vicovaro, Riccardo Boscariol, Mario Dalmaso

**Affiliations:** 1 Department of General Psychology, University of Padova, Via Venezia 8, 35131 Padova, Italy; 2 Department of Developmental and Social Psychology, University of Padova, via Venezia 8, 35131 Padova, Italy

**Keywords:** SNARC, SNARC-like effect, Visual speed, Spatial representation

## Abstract

Numerical and nonnumerical magnitudes can be represented along a hypothetical left-to-right continuum, where smaller quantities are associated with the left side and larger quantities with the right side. However, these representations are flexible, as their intensity and direction can be modulated by various contextual cues and task demands. In four experiments, we investigated the spatial representation of visual speed. Visual speed is inherently connected to physical space and spatial directions, making it distinct from other magnitudes. With this in mind, we explored whether the spatial representation of visual speed aligns with the typical left-to-right orientation or is influenced dynamically by the movement direction of the stimuli. Participants compared the speed of random dot kinematograms to a reference speed using lateralised response keys. On each trial, all dots moved consistently in one single direction, which varied across the experiments and could also vary from trial to trial in Experiments 2 and 4. The dot movements were left-to-right (Experiment 1), random across a 360° spectrum (Experiment 2), right-to-left (Experiment 3), and random left-to-right or right-to-left (Experiment 4). The results supported a relatively stable left-to-right spatial representation of speed (Experiments 1–3), which was compromised by mutable motion directions along the horizontal axis (Experiment 4). We suggest that representing stimuli as belonging to a single set rather than different sets, may be crucial for the emergence of spatial representations of quantities.

## Introduction

In Western cultures, relatively small and large numerosities are typically associated with the left and the right side of space, respectively. A classic experimental demonstration of this association is provided by the Spatial-Numerical Association of Response Codes (SNARC) effect. It consists in the fact that, when participants are tasked with classifying integers as even or odd using two lateralised response keys (i.e., a parity judgment task), faster responses typically emerge when relatively small numbers are responded with a left-side key and relatively large numbers are responded with a right-side key, compared with when the opposite occurs (Dehaene et al., 1993). The SNARC effect also emerges when participants are explicitly asked to classify the magnitude of integers as smaller or bigger than a reference number (i.e., a magnitude comparison task; Dehaene et al., 1990). As a possible explanation of the SNARC effect, Dehaene et al. (1993) suggested that numbers would be represented in the cognitive system along a horizontal line that is oriented from left to right (i.e., the mental number line; for alternative accounts of the SNARC, see Gevers et al., 2006; Proctor & Cho, 2006; van Dijck & Fias, 2011).

Space–number associations appear to be deeply shaped by cultural habits, like number reading direction (Dehaene et al., 1993; Hung et al., 2008; Shaki et al., 2009; Toomarian & Hubbard, 2018; but see Pitt & Casasanto, 2020) and finger counting direction (Fischer, 2008; Fischer & Brugger, 2011; Pitt & Casasanto, 2020). Moreover, converging evidence indicates that the SNARC effect is a relatively flexible and malleable phenomenon, shaped by various contextual factors and task demands. For example, a reversed SNARC effect was observed when participants were directed to link smaller and larger numbers with the right and left sides of the space, respectively (Notebaert et al., 2006). This reversal also manifested when numbers were mapped onto an analogical clock as time measures (Bächtold et al., 1998) or onto the numeric keypad of a smartphone (Mingolo et al., 2021). Crucially, in both setups, the arrangement placed smaller/larger numbers to the right/left, respectively. Similarly, Fischer et al. (2010) identified a null or reversed SNARC effect following a parity judgment task that was preceded by reading a text where small and large numbers were situated on the right and left sides of the space. Additionally, a null or reversed SNARC pattern emerged when the number classification task followed the presentation of decreasing number sequences (Ginsburg & Gevers, 2015; Lindemann et al., 2008; van Dijck & Fias, 2011). Pitt and Casasanto (2020) found a decreased SNARC effect when a parity judgment task followed a training session where participants counted with their fingers from right to left. Moreover, it has been demonstrated that space–number associations in parity judgment tasks can be rapidly recalibrated from trial to trial (Gökaydin et al., 2018; Pfister et al., 2013), underscoring the dynamic and adaptable nature of these effects.

Several authors have interpreted the flexibility of the SNARC effect as evidence against Dehaene et al.’s (1993) claim that the SNARC reflects the existence of a stable long-term association between numbers and space. These authors have suggested that the SNARC effect would stem from the use of short-term task-dependent strategies aimed at facilitating the encoding of numbers in specific contexts (Fischer, 2006; Gevers et al., 2006; Myachykov et al., 2014; Shaki & Fischer, 2018; Sixtus et al., 2019). Despite this, support for the existence of a deep link in the brain between left space and small quantities and between right space and large quantities comes from neuropsychological (Vuilleumier et al., 2004; Zorzi et al., 2002, 2006), developmental (e.g., de Hevia et al., 2017; Di Giorgio et al., 2019), and animal cognition (e.g., Rugani et al., 2015) studies. In addition, some recent theories suggest that space–number associations may stem from hemispheric asymmetries regulating approach/withdrawal behaviours (Vallortigara, 2017) and/or the processing of low and high spatial frequencies (Felisatti et al., 2020).

According to Treccani and Umiltà (2011), the documented adaptability of the SNARC effect does not inherently contradict the notion of a stable space–number association. In fact, the adaptable space–quantity associations arising from specific contexts and task demands might engage response recoding strategies that overlay upon the underlying stable left-to-right representation. Guida and Campitelli (2019) suggested that, depending on the characteristics of the task, the spatial representation of numbers can rely on flexible and task-dependent strategies based on the activation of working memory, or more stable representations stored in long-term memory. Theoretically, these two concepts are not mutually exclusive, although, according to Guida and Campitelli (2019), only one type of spatial representation would be active at a time.

These propositions gain further support from the observation that neutralising or reversing the classic SNARC effect necessitates precise experimental interventions and constraints. For instance, Ginsburg and Gevers (2015) found that altering the relative order of numbers within a sequence presented prior to the number classification task only disrupted the conventional left-to-right representation when the sequence order needed to be actively retained in working memory during the main task. Similarly, Mingolo et al. (2024) documented a reversed SNARC effect when participants had to decide whether a number is associated with the left or right of an analogical clock face, where, as already mentioned, small and large numbers conventionally associate with the right and left sides, respectively. However, a classic SNARC effect emerged when participants were solely exposed to a clock face representation before executing a classic parity judgment or magnitude comparison task. This implies that alternative space–number associations can supplant the left-to-right representation solely when specifically pertinent to the task. Pitt and Casasanto (2020) found that the strength of the SNARC effect is influenced by finger-counting habits. Nevertheless, it is important to emphasise that intensive training in finger counting, involving repeated counting from right to left, merely diminished the intensity of the left-to-right SNARC effect without fully neutralising or reversing it.

In summary, the available empirical evidence suggests that, in Western cultures, the left-to-right representation of numbers remains a fairly resilient phenomenon. Any nullification or reversal of this established representation seems achievable only through deliberate experimental manipulations that accentuate the prominence of the opposing representation.

### Beyond numbers: From SNARC to SNARC-like effects

Interestingly, space–quantity associations appear to exist beyond the domain of numbers. Indeed, SNARC-like effects have been reported for a variety of nonnumerical magnitudes (for a meta-analysis and review, see Macnamara et al., 2018), like size (Prpic et al., 2020; Ren et al., 2011; Sellaro et al., 2015), luminance (Fumarola et al., 2014; Ren et al., 2011; Wang et al., 2022), pitch height (Lidji et al., 2007; Rusconi et al., 2006), loudness (Chang & Cho, 2015; Hartmann & Mast, 2017), music notation (Fumarola et al., 2020; Prpic et al., 2016), time (Ishihara et al., 2008; Mariconda et al., 2022; Vallesi et al., 2008; Weger & Pratt, 2008; Zhao et al., 2018), music tempo (De Tommaso & Prpic, 2020; Mariconda et al., 2024b), face age (Dalmaso et al., 2023a; Dalmaso & Vicovaro, 2021), and weight (Dalmaso & Vicovaro, 2019). The results of these studies, mainly conducted with Western participants, generally indicate that small and large magnitudes are associated with the left and the right side of space, respectively.^1^

The similarity between SNARC and SNARC-like effects has inspired theories according to which the processing of numerical and nonnumerical would share common underlying mechanisms (e.g., a theory of magnitude, Walsh, 2003, 2015; the mental magnitude line, Holmes & Lourenco, 2011). According to these theories, the similarity between the spatial representation of numerical and nonnumerical magnitudes would be related to the functional characteristics of a common underlying system for magnitude processing (Toomarian & Hubbard, 2018). In contrast to this viewpoint, Casasanto and Pitt (2019; see also Pitt & Casasanto, 2022) have put forth a series of points supporting the idea that SNARC-like effects might not necessarily indicate the spatial representation of magnitude, but rather the spatial organisation of ordinality. One of their key arguments stems from the observation that SNARC-like effects have been observed in contexts involving categories of elements where the concept of magnitude does not apply. Notable examples include valence-related stimuli (see Footnote 1), months of the year, and alphabet letters (Gevers et al., 2003).

Relatively few studies have directly explored the factors that can influence the intensity and orientation of SNARC-like effects, with the majority of these studies concentrating on the temporal dimension. Torralbo et al. (2006), found a sagittal back-to-front representation of temporally ordered events when these were classified verbally by the participants, whereas a horizontal left-to-right representation emerged when the same classification task was performed through lateralised response keys. According to the authors, the spatial representation of time would flexibly adapt to the most salient spatial dimension activated through the experimental task. In another study on the spatial representation of time, Casasanto and Bottini (2014) found a left-to-right representation of time-related sentences when these were written in standard orthography, and a reversed right-to-left representation for sentences written in mirror-reversed orthography, suggesting that reading direction can shape the spatial representation of time (see also Pitt & Casasanto, 2020; for contrary evidence, see Beracci et al., 2022a, 2022b). Pitt and Casasanto (2020) also found that the strength of the space–time association was modulated by finger counting training in which participants were instructed to count down to the right or to the left. Beyond the time domain, Wang et al. (2022) found a SNARC-like effect consistent with a left-to-right representation of luminance when participants compared the luminance of target squares with that of a reference square. However, no SNARC-like effect emerged when participants judged the luminance of left and right arrows instead of squares. This suggests that the directional information the stimuli convey can interfere with the spatial representation of nonnumerical magnitudes.

Evidence that supports the malleable nature of SNARC-like effects also comes from studies that have explored the representation of nonnumerical magnitudes along the vertical dimension of space. For instance, Vicovaro and Dalmaso (2021) found that weight could be represented either from bottom-to-top or from top-to-bottom, depending on whether weight was approached conceptually or related to the actual weights of objects that participants had to weigh before a classification task. Similarly, Dalmaso et al., (2023b) found that the way time-related stimuli were spatially represented depended on whether they were grouped into distinct categories or treated as part of a continuous range.

### A SNARC-like effect for visual speed? Outline of the present work

The present set of experiments investigates a novel area, examining the potential presence of a SNARC-like effect associated with visual speed. Visual speed has a distinctive spatial quality due to its intrinsic link with motion, which often corresponds to specific spatial directions. This raises intriguing questions: Does the cognitive system spatially map an inherently spatial and directional quantity? Is this mapping constrained to the usual left-to-right pattern, or does it adapt to the direction of the motion?

A notable aspect of visual speed sets it apart from numerical values and other dimensions—it allows exploration of the possible flexibility of its spatial associations without requiring contrived tasks or experimental manipulations. For instance, it enables investigation into whether a left-to-right or right-to-left space–speed association emerges based on motion direction. Under the hypothesis of stable left-to-right magnitude representations, as posited within the framework of the mental number line (Dehaene et al., 1993) and mental magnitude line (Holmes & Lourenco, 2011), relatively slow and fast speeds are expected to be associated with the left and right sides of space, respectively, regardless of the motion direction characterising the visual stimuli. Conversely, based on the hypothesis that space–magnitude associations are flexible and arise from short-term, task-dependent strategies designed to facilitate magnitude encoding in specific contexts (Fischer, 2006; Gevers et al., 2006; Myachykov et al., 2014; Shaki & Fischer, 2018; Sixtus et al., 2019), the direction of stimulus motion may serve as an external cue that dynamically influences the direction of space–magnitude associations. Within this framework, the cognitive system is thought to adapt spatial associations flexibly to align with contextual cues. Thus, for example, when visual stimuli move consistently in a right-to-left direction, this motion direction could directly shape the spatial representation, leading to a corresponding right-to-left mapping of visual speed. To address these research questions, we devised four experiments to reveal the spatial representation of visual speed and its interplay with motion direction. Hypotheses and methods were preregistered (https://aspredicted.org/W13_NJ6).^2^

In all experiments, participants observed moving stimuli on a plane parallel to their frontal view in each trial. Their task was to categorise the observed speed as either slower or faster than a reference speed by pressing either a left-side key or a right-side key. In Experiment 1, stimuli moved left to right. An anticipated outcome is the emergence of a left-to-right representation of visual speed, with slower speeds mapped on the left and faster speeds mapped on the right. This would be consistent with both the hypothesis that speed–space associations are adaptable to motion direction and the hypothesis that they reflect stable left-to-right space–magnitude associations. At a behavioural level, this spatial representation would manifest as a SNARC-like effect, wherein faster responses are expected with the left key than with the right key for slow speeds, and vice versa for fast speeds.

Experiment 2 tested the generalizability of the effect in a more neutral framework wherein the motion direction of the stimuli was randomly determined across a 360° spectrum from one trial to another. Unlike Experiment 1, the trial-by-trial variability in motion direction precluded any consistent visual cue for the spatial representation of speed along a specific direction. Under the hypothesis of flexible, task-dependent space–magnitude associations, the absence of external cues that could potentially trigger representations along a specific direction may lead to the emergence of a standard left-to-right representation, possibly influenced by long-standing cultural habits like reading direction. According to the hypothesis of stable left-to-right space–magnitude representations, a standard SNARC-like effect akin to Experiment 1 should persist despite the altered context.

Experiment 3 aimed to critically test the flexibility or stability of the effect. In each trial, stimuli consistently moved from right to left. We hypothesised that, if the effect could be influenced by the directional information provided by the stimuli, a reversed right-to-left effect might emerge. Conversely, if a left-to-right association persisted despite the challenging context, it would support the stability and reliability of the left-to-right representation.

Lastly, Experiment 4 was devised to offer a deeper exploration of the dynamic versus steadfast nature of the effect. While adhering to the horizontal axis motion, akin to Experiments 1 and 3, each trial introduced an element of randomness to the motion direction—alternating between left-to-right and right-to-left. Based on the hypothesis suggesting that the effect is influenced by the motion direction of the target stimuli, the opposing left-to-right and right-to-left directions could potentially counterbalance one another, resulting in the absence of a clearly defined spatial representation of visual speed (i.e., the absence of a SNARC-like effect). According to the hypothesis of stable left-to-right space–magnitude representations, a standard SNARC-like effect should persist, despite the conflicting visual cues.

## Experiment 1

### Method

#### Participants

The determination of the sample size adhered to the guidelines outlined by Brysbaert and Stevens (2018) for linear mixed-effects models incorporating subjects and items as random effects. These guidelines, which refer to reaction time studies characterised by typically subtle effect sizes, advocate accumulating a minimum of 1,600 data points per experimental condition to ensure adequate statistical power. Our experimental design was a 2 (response side: left or right) × 2 (target speed: slower or faster than the reference) within-subjects factorial design. Because we planned to collect 60 trials per condition, we needed at least 27 participants to achieve adequate statistical power (60 × 27 = 1,620). As a measure of caution, in accordance with our preregistered analysis plan, we included an additional three participants.

The sample was composed of 30 students (*M*_age_ = 23.63 years, *SE* = 0.45, six men) at the University of Padova. They took part in exchange for course credits. Five of them self-identified as left-handed. Manual preference was also assessed through the Edinburgh Handedness Inventory (EHI) short form (Veale, 2014), a four-item survey that produces a score ranging from − 100 to + 100, reflecting left to right-hand preference. The participants had an average EHI score of 60.98 (*SE* = 13.17, range: − 100–100). The EHI scores aligned with self-declared handedness. All five participants who identified as left-handed scored below − 40 and were therefore categorised as left-handed. Similarly, those with scores exceeding 40 were consistently identified as right-handed.

This study was approved by the Ethics Committee for Psychological Research at the University of Padova (approval number: 4480) and conducted following the ethical standards of the 1964 Helsinki Declaration. All participants read and signed a written informed consent.

#### Apparatus

The participants were tested individually in a quiet, comfortable room. The script for the experiment was created with PsychoPy3 (Peirce et al., 2022), and the experiment itself was presented on a 35.54-cm × 20-cm LCD screen (refresh rate 60 Hz, screen resolution 1,366 × 768 pixels). Participants sat about 50 cm from the screen, the background of which was grey. A standard keyboard, placed centrally with respect to the screen, was used to record the participants’ manual responses.

**Fig. 1:**
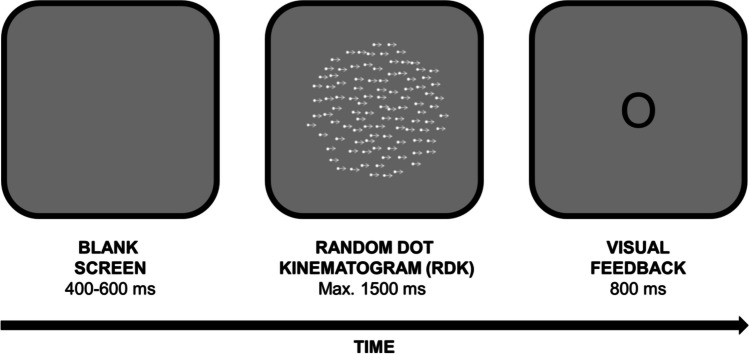
Illustration of a trial and the stimuli (not drawn to scale) used in the experiments. The arrows depicted in the random dot kinematogram (RDK) representation indicate motion direction (in this example, from left to right) and have been added to this figure solely for illustrative purposes. The visual feedback ‘O’ indicated a correct response

The stimuli employed consisted of random dot kinematograms (RDKs; see Fig. 1) generated using the *Dots* tool within PsychoPy3 Builder framework. RDKs, a well-established visual stimulus arrangement, consist of dots that navigate within a designated area at a specified velocity. The ensuing attributes of RDKs remained unaltered throughout the experiment: dimension of the area of navigation (circular shape, 10 cm in diameter), time-out duration (1.5 s; the specific duration depended on participant’s response), dot count (100), motion direction of dots (left-to-right), lifespan of dots (60 frames, 1 s), dot size (5 pixels), and dot colour (white). It is noteworthy that the dot count pertains to the number of dots concurrently displayed on the screen with each frame. Each dot remained visible for a maximum of 60 frames (1 s), after which it was replaced by a dot positioned at a random location within the RDK area. If a dot traversed beyond the confines of the RDK boundary before 60 frames had elapsed, it was replaced by a new dot in a random position within the designated RDK area. This systematic replacement ensured the constancy of dot count per frame throughout the entire stimulus duration.

It is important to underline that Experiment 1 exclusively entailed the horizontal motion of dots, strictly adhering to the left-to-right trajectory as depicted in Fig. 1. Within each trial, the entire set of dots constituting the RDK maintained uniform motion, while the specific speed of the entire set of dots varied from one trial to another. A reference speed of 8 cm/s was established, serving as a benchmark against which 20 distinct target speeds were compared. Ten of these target speeds were slower than the reference speed, spanning from 5.5 to 7.75 cm/s in increments of 0.25 cm/s. The remaining ten target speeds exceeded the reference speed, ranging from 8.25 to 10.5 cm/s, with analogous increments of 0.25 cm/s.

The stimulus configuration based on RDKs allowed us to prevent possible confounds between speed and other potentially correlated dimensions, particularly motion duration and travel distance. Indeed, if a single dot moving along a fixed distance were used, its speed and duration would be perfectly correlated (i.e., higher speed – shorter duration, lower speed – longer duration). Similarly, with a single dot moving for a fixed duration, its speed would be perfectly correlated with travel distance (i.e., higher speed – longer distance, lower speed – shorter distance). By using RDKs, in which different dots move over varying distances and durations depending on their starting positions within the circular area, we could counteract the correlations of speed with the other two dimensions.

#### Procedure

Participants were instructed via on-screen instructions (black Arial font, height = 0.07 norm units) that they would be presented with moving dots. Their task was to judge whether the speed of the dots was slower or faster than a reference speed. They were directed to place their left index finger on the ‘A’ key and their right index finger on the ‘L’ key, using these keys to classify the target speed as slower or faster than the reference speed. The mapping between response category (‘slower’ or ‘faster’) and response key (‘A’ or ‘L’) was counterbalanced across participants. Participants were instructed to respond as quickly and accurately as possible after the dots appeared on the screen.

At the beginning of the experiment, participants pressed the space bar to view the reference speed. The RDK corresponding to the reference speed (8 cm/s) was then presented, preceded and followed by a 500-ms blank screen. Subsequently, participants pressed the space bar again to view the reference speed for a second time. Then, participants were informed that they would engage in some practice trials and were reminded of the correct response-key associations. They were asked to initiate the practice block by pressing the space bar. Each practice trial started with a blank screen lasting for a randomly determined duration between 400 and 600 ms, followed by the presentation of an RDK featuring one randomly selected target speed. Following a response (or after 1,500 ms elapsed without a response) visual feedback (black Arial font, height = 0.07 norm units) was displayed on the screen for 800 ms. This was an ‘O’ symbol for correct responses, an ‘X’ symbol for incorrect responses and the phrase ‘TOO SLOW’ if no response was provided (see Fig. 1). Subsequently, a new practice trial started. In the practice block, all 20 target speeds were presented once in a random order. Responses to the practice trials were not analysed.

At the conclusion of the first practice block, participants were informed that the experimental block would be started. They were reminded to respond as quickly and as accurately as possible and were prompted to press the space bar to view the reference speed, which was presented twice as it was at the beginning of the practice block. The trials within the first experimental block followed the same structure as those in the first practice block. In the first experimental block, 120 experimental trials were presented in a random order, derived from six repetitions of the 20 target speeds. After every 30, 60, and 90 experimental trials, participants were again shown the reference speed using the same procedure as described before.

At the conclusion of the first experimental block, participants were given instructions identical to those given before the first practice block, except that the association between response key and response category was reversed. Subsequently, participants completed a second practice block and a second experimental block. A total of 240 experimental trials were presented, with 120 trials in each of the two experimental blocks.

### Results and discussion

The main analyses were performed on the reaction times (RTs) of correct responses. Wrong responses (15.17% of trials) were removed and analysed separately, and no statistically significant effects emerged.^3^ Importantly, all the participants performed well above the chance level, with a maximum percentage of individual errors of 22.1%. Missed responses (0.51% of trials) were removed and not analysed further due to their low percentage. Outlier responses, defined as correct trials with RTs three standard deviations above or below the participant’s mean, were also removed (1.63% of trials).

In accordance with the preregistered analysis plan, RTs for correct responses were analysed using linear mixed-effects models (R package *lme4*; Bates et al., 2015). The fixed effects were response side (left or right key), the relative speed of the target (slower or faster than the reference), and the interaction. Speed was treated as a dichotomous variable, consistent with the assumption that in explicit magnitude classification tasks, the relationship between target magnitude and RTs follows a step function rather than a linear one (Gevers et al., 2006). The models differed only in their random components. Depending on the specific model, random effects could include the by-subject intercept, the by-item intercept, the by-subject slope for speed, the by-subject slope for response side, and the by-item slope for response side. In this context, ‘item’ refers to absolute speed, with 20 items corresponding to the 20 target speeds. Increasingly complex models, characterised by an increasing number of random effects, were compared using a likelihood ratio test.

**Fig. 2:**
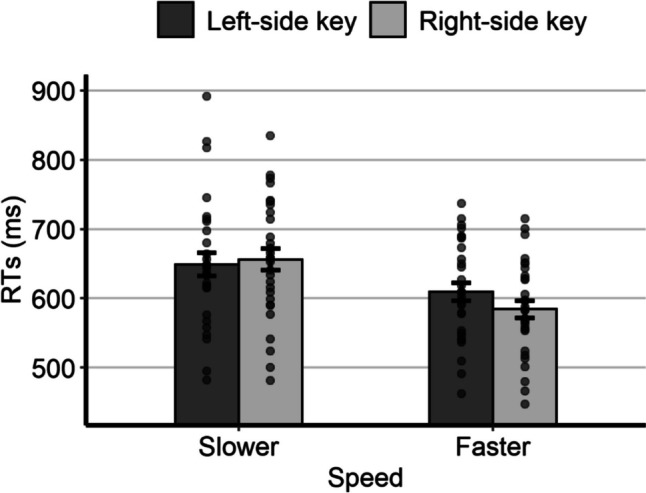
Mean individual RTs of Experiment 1 as a function of relative speed and response side. Error bars are *SEM*

The model with the best fit to the data had, as random effects, the by-subject intercept, the by-subject slope for speed, the by-subject slope for response side, and the by-item intercept. This model was then submitted to a Type 3 analysis of variance (ANOVA) with Satterthwaite’s approximation for the degrees of freedom (R package *lmerTest*; Kuznetsova et al., 2017), which is suitable for the analysis of linear mixed-effects models. Post hoc tests were performed through the R package *lsmeans* (Lenth, 2016). The mean individual RTs as a function of relative speed and response side are represented in Fig. 2. The main effect of relative speed was statistically significant, *F*(1,23.3) = 17.63, *p* < 0.001, due to shorter RTs for the fast speed (*M* = 599 ms, *SE* = 14.9) than for the slow speed (*M* = 657 ms, *SE* = 17.1). The main effect of response side was also statistically significant, *F*(1,29.4) = 4.57, *p* = 0.041, due to shorter RTs with the right key (*M* = 623 ms, *SE* = 14.5) than with the left key (*M* = 633 ms, *SE* = 14.8). The interaction was also statistically significant, *F*(1,5877.6) = 19.15, *p* < 0.001, thus indicating the presence of a SNARC-like effect. Post hoc comparisons showed that, for the slow speed, the RTs were slightly shorter with the left key (*M* = 655 ms, *SE* = 17.6) than with the right key (*M* = 660 ms, *SE* = 17.0), although the difference was not statistically significant (*p* = 0.389). For the fast speed, the RTs were significantly shorter with the right key (*M* = 586 ms, *SE* = 15.2) than with the left key (*M* = 612 ms, *SE* = 17.0; *p* < 0.001).

The results indicate the existence of a SNARC-like effect for visual speed. The direction of the interaction (Fig. 2) is consistent with the hypothesis that, in the case of stimuli moving along a left-to-right direction, relatively slow and fast speeds were represented on the left and on the right side of space, respectively. However, it is worth noting that the difference between the RTs with the left and the right key was clearly more pronounced for the fast speeds than for the slow speeds, a result on which we will return in the General Discussion.

For completeness, we also explored the possible existence of a distance effect, which refers to a well-known phenomenon by which, in magnitude comparison tasks, the absolute RTs tend to decrease as the absolute difference between the magnitude of the target and the magnitude of the reference tends to increase (i.e., responses are faster for large absolute differences in magnitude between the target and the comparison; see e.g., Dalmaso & Vicovaro, 2019; Gevers et al., 2006; Moyer & Landauer, 1967). The possible distance effect was analysed through a linear mixed effects model with the RTs as the dependent variable, the absolute difference between the speed of the target and the speed of the reference as a continuous fixed effect, the by-subject intercept and slope as the random effects. The negative regression coefficient, which was significantly different from zero, indicates the existence of a distance effect, *b* = − 0.035, *SE* = 0.004, *t*(29.2) = − 8.77, *p* < 0.001.

## Experiment 2

It can be argued that a SNARC-like effect for visual speed emerged in Experiment 1 because the motion direction of the stimuli (left-to-right) served as a cue for the spatial representation of target magnitude. Experiment 2 aimed to test the generalizability of the results of Experiment 1 in a context where the motion direction of the stimuli varied randomly across the full range of possible directions (360°). Specifically, we sought to determine if a SNARC-like effect for visual speed could emerge even when the motion direction of the stimuli did not provide any consistent cues for the direction of the spatial representation of visual speed along the horizontal axis.

### Method

#### Participants

The criteria for determining the sample size were the same as in Experiment 1. Thirty students at the University of Padova took part in this experiment in exchange for course credits (*M*_age_ = 23.03 years, *SE* = 0.41, six men). None of them had participated in Experiment 1. The participants had an average EHI score of 70.08 (*SE* = 10.8; range: − 100–100). The EHI scores tended to align with self-declared handedness. Three participants who identified as left-handed scored below − 40 and were therefore categorised as left-handed, whereas one participant who identified as left-handed scored 0 and was therefore classified as ambidextrous. Participants with scores exceeding 40 were consistently identified as right-handed.

This study was approved by the Ethics Committee for Psychological Research at the University of Padova (approval number: 4480) and conducted following the ethical standards of the 1964 Helsinki Declaration. All participants read and signed a written informed consent.

#### Apparatus

The apparatus was the same as in Experiment 1.

#### Procedure

The procedure was the same as in Experiment 1, except that, on each trial, the direction of the dots in the RDKs varied randomly from 0° to 360° (in each trial, all dots always moved in the same randomly chosen direction). It is important to note that the motion direction in the RDKs representing the reference speed was selected randomly, as was the motion direction in the RDKs representing the target speed. However, the motion direction remained the same across the two successive presentations of the reference speed.

### Results and discussion

The data were analysed as in Experiment 1. Wrong responses (18.22% of trials) were removed and analysed separately, and no statistically significant effects emerged.^4^ All the participants performed above the chance level, with a maximum percentage of individual errors of 32.1%. Missed responses (0.86% of trials) were removed and not analysed further due to their low percentage. Outlier responses, defined as correct trials with RTs three standard deviations above or below the participant’s mean, were also removed (1.29% of trials).

**Fig. 3:**
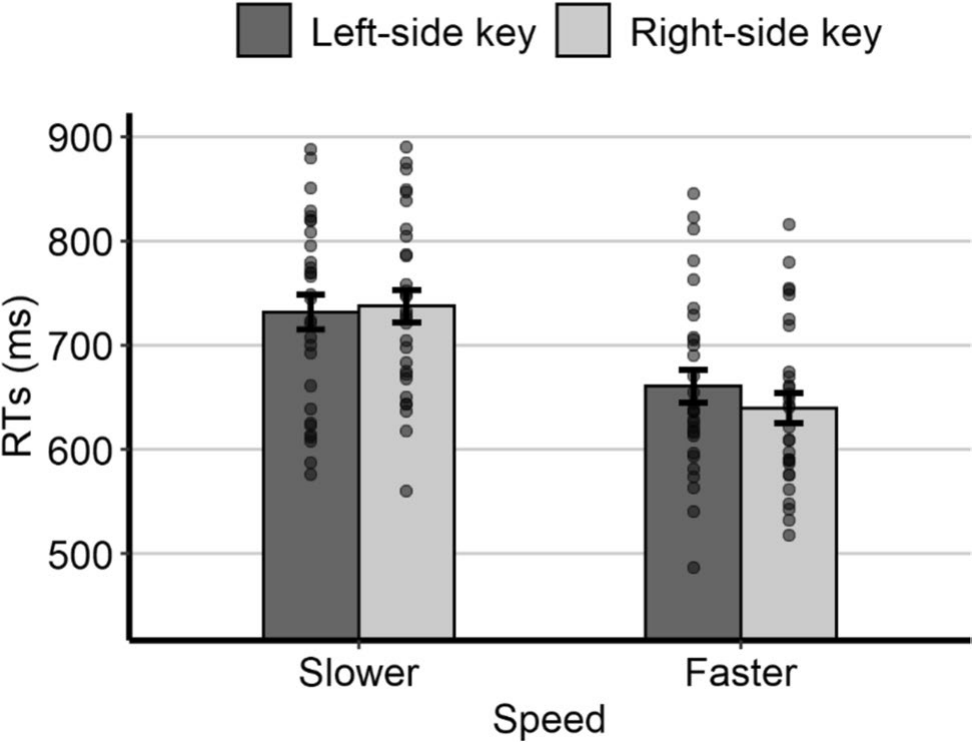
Mean individual RTs of Experiment 2 as a function of relative speed and response side. Error bars are *SEM*

The mean individual RTs as a function of relative speed and response side are represented in Fig. 3. As for the analysis of the possible SNARC-like effect, the model with the best fit to the RTs had, as fixed effects, the response side, the relative speed, and the interaction; as random effects it had the by-subject intercept, the by-subject slope for response side, and the by-item intercept. The main effect of relative speed was statistically significant, *F*(1,23.5) = 32.34, *p* < 0.001, due to shorter RTs for the fast speed (*M* = 652 ms, *SE* = 16.6) than for the slow speed (*M* = 740 ms, *SE* = 17.5). The main effect of response side was not statistically significant, *F*(1,5674.9) = 3.22, *p* = 0.073, despite slightly shorter RTs with the right key (*M* = 693 ms, *SE* = 15.4) than with the left key (*M* = 700 ms, *SE* = 15.4). Importantly, the interaction was statistically significant, *F*(1,5677.4) = 10.05, *p* = 0.002, thus indicating the presence of a SNARC-like effect. Post hoc comparisons showed that, for the slow speed, the RTs were slightly shorter with the left key (*M* = 738 ms, *SE* = 17.8) than with the right key (*M* = 743 ms, *SE* = 17.8), although the difference was not statistically significant (*p* = 0.347). For the fast speed, the RTs were significantly shorter with the right key (*M* = 642 ms, *SE* = 16.9) than with the left key (*M* = 662 ms, *SE* = 16.9; *p* < 0.001).

As for the distance effect of Experiment 2, the model with the best fit with the data had the absolute difference between the speed of the target and the speed of the reference as a fixed effect, the by-subject intercept and slope as the random effects. The negative regression coefficient, which was significantly different from zero, indicates the existence of a distance effect, *b* = − 0.036, *SE* = 0.004, *t*(29.2) = − 8.19, *p* < 0.001.

To directly compare the results of Experiment 2 with those of Experiment 1, a between-subject ‘experiment’ factor was included as a fixed effect in the model that proved to have the best fit to the data of Experiment 2. Therefore, the model had, as fixed effects, the experiment, the response side, the relative speed, and all the interactions; as random effects it had the by-subject intercept, the by-subject slope for response side, and the by-item intercept. Here we focus solely on the main and interaction effects involving the experiment factor. There was a statistically significant main effect of experiment, *F*(1,58) = 12.83, *p* < 0.001, due to shorter RTs in Experiment 1 (*M* = 629 ms, *SE* = 14.9) than in Experiment 2 (*M* = 696 ms, *SE* = 14.9). There was also a statistically significant two-way interaction between experiment and relative speed, *F*(1,57.6) = 8.99, *p* = 0.004. In both experiments, the RTs were shorter for the fast than for the slow speed; however, the difference was more pronounced in Experiment 2 (*M* = − 87.5 ms, *SE* = 14.7, *p* < 0.001) than in Experiment 1 (*M* = − 58.9 ms, *SE* = 14.7, *p* < 0.001). The interaction between experiment and response side was not statistically significant, *F*(1,11,588.6) = 0.33, *p* = 0.565, and, importantly, the three-way interaction was not statistically significant, *F*(1,11,591.9) = 0.20, *p* = 0.656. The latter result indicates the lack of a statistically significant difference in the magnitude of the SNARC-like effect that emerged from the two experiments.

The longer RTs in Experiment 2 compared with Experiment 1 suggest that the magnitude comparison task was more challenging when the dots moved randomly rather than in a fixed direction, a hypothesis also confirmed by the higher mean percentage of errors in the second experiment. Despite these differences in stimulus characteristics and task difficulty, similar SNARC-like (and distance) effects were observed in both experiments. These results are consistent with the hypothesis of a consistent left-to-right representation of visual speed independent of motion direction. However, these results are insufficient to reject the hypothesis that the motion direction of the stimuli can influence the spatial representation of visual speed. In Experiment 1, there was a perfect overlap between the left-to-right representation associated with cultural habits and the motion direction of the stimuli. In Experiment 2, the lack of a consistent motion direction may have allowed the standard left-to-right representation to emerge. Therefore, a stronger test of the flexibility versus the stability of the spatial representation of visual speed involves a task in which the two spatial vectors (the left-to-right vector associated with cultural habits and the vector associated with the motion of the dots) are directly contrasted. In the next experiment, we presented participants with dots moving from right to left.

## Experiment 3

The third experiment aimed to provide a more direct test of the two main hypotheses guiding this work. In this experiment, all the dots in the RDKs moved consistently from right to left. This direction is opposite to the ‘default’ left-to-right direction that typically characterises the spatial representation of magnitudes in Western participants. Under the hypothesis that SNARC-like effects reflect highly flexible and task-dependent strategies designed to facilitate stimulus encoding, it can be predicted that in a context where participants are exclusively exposed to stimuli moving from right to left, the spatial representation of the target magnitude may align with this orientation, with relatively slow speeds represented on the right and relatively fast speeds on the left. Conversely, under the hypothesis that SNARC-like effects reflect a stable association between small magnitudes and left space, and large magnitudes and right space—an association potentially related to evolutionarily ancient brain asymmetries (see Felisatti et al., 2020; Vallortigara, 2017)—a left-to-right spatial representation of visual speed may emerge despite the context.

### Method

#### Participants

The criteria for determining the sample size were the same as in the previous experiments. Thirty students at the University of Padova took part in this experiment in exchange for course credits (*M*_age_ = 23.6 years, *SE* = 0.56, nine men). None of them had participated in Experiments 1 or 2. The participants had an average EHI score of 76.11 (*SE* = 10.51; range: − 100–100). The EHI scores tended to align with self-declared handedness. All three participants who identified as left-handed scored below − 40. Of the 27 participants who identified as right-handed, two scored between − 40 and 40 and were therefore classified as ambidextrous. Participants with scores exceeding 40 were consistently identified as right-handed.

This study was approved by the Ethics Committee for Psychological Research at the University of Padova (approval number: 4480) and conducted following the ethical standards of the 1964 Helsinki Declaration. All participants read and signed a written informed consent.

#### Apparatus

The apparatus was the same as in Experiment 1.

#### Procedure

The procedure was the same as in Experiment 1, except that, on each trial, the motion direction of the dots in the RDKs was right-to-left.

### Results and discussion

The data were analysed as in Experiment 1. Wrong responses (16.49% of trials) were removed and analysed separately, and the results are consistent with those of the RTs analysis.^5^ All the participants performed above the chance level, with a maximum percentage of individual errors of 29.2%. Missed responses (0.47% of trials) were removed and not analysed further due to their low percentage. Outlier responses, defined as correct trials with RTs three standard deviations above or below the participant’s mean, were also removed (1.52% of trials).

**Fig. 4:**
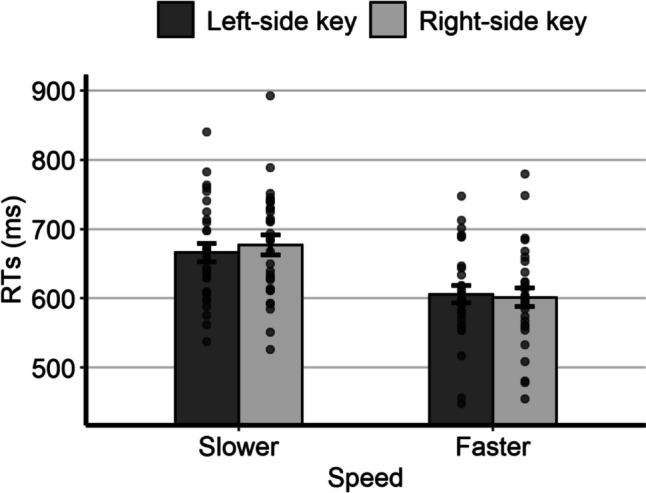
Mean individual RTs of Experiment 3 as a function of relative speed and response side. Error bars are *SEM*

The mean individual RTs as a function of relative speed and response side are represented in Fig. 4. As for the analysis of the possible SNARC-like effect, the model with the best fit to the RTs had, as fixed effects, the response side, the relative speed, and the interaction; as random effects it had the by-subject intercept, the by-subject slope for response side, the by-subject slope for the relative speed, and the by-item intercept. The main effect of relative speed was statistically significant, *F*(1,19.7) = 19.84, *p* < 0.001, due to shorter RTs for the fast speed (*M* = 606 ms, *SE* = 16.2) than for the slow speed (*M* = 679 ms, *SE* = 16.5). The main effect of response side was not statistically significant, *F*(1,29.1) = 0.60, *p* = 0.446, despite slightly shorter RTs with the left key (*M* = 641 ms, *SE* = 13.9) than with the right key (*M* = 645 ms, *SE* = 15.0). Importantly, the interaction was statistically significant, *F*(1,5801.3) = 6.57, *p* = 0.010, thus indicating the presence of a SNARC-like effect. Post hoc comparisons showed that, for the slow speed, the RTs were significantly shorter with the left key (*M* = 672 ms, *SE* = 16.3) than with the right key (*M* = 685 ms, *SE* = 17.4; *p* = 0.035). For the fast speed, the RTs were slightly shorter with the right key (*M* = 604 ms, *SE* = 16.9) than with the left key (*M* = 609 ms, *SE* = 16.0), although the difference was not statistically significant (*p* = 0.351). Interestingly, the direction of the interaction is consistent with the hypothesis of a left-to-right representation of visual speed (see Fig. 4).

As regards the distance effect, the model with the best fit with the data had the absolute difference between the speed of the target and the speed of the reference as a fixed effect, the by-subject intercept and slope as the random effects. The statistically significant negative regression coefficient confirms the existence of a distance effect, *b* = − 0.040, *SE* = 0.004, *t*(29.11) = − 9.53, *p* < 0.001.

We also directly compared the results of Experiment 3 and Experiment 1. The model had, as fixed effects, the experiment, the response side, the relative speed, and all the interactions; as random effects it had the by-subject intercept, the by-subject slope for response side, the by-subject slope for the relative speed, and the by-item intercept. We focus solely on the main and interaction effects of the experiment factor. The only statistically significant effect was the two-way interaction between experiment and response side, *F*(1,58.5) = 4.13, *p* = 0.047, because, in Experiment 1, the RTs were significantly shorter with the right-side key (*M* = 624 ms, *SE* = 14.7) than with the left-side key (*M* = 634 ms, *SE* = 14.3, *p* = 0.035), whereas in Experiment 3 the RTs were slightly shorter with the left-side key (*M* = 640 ms, *SE* = 14.3) than with the right-side key (*M* = 644 ms, *SE* = 14.7), although the difference was not statistically significant (*p* = 0.445). The three-way interaction was not statistically significant, indicating that a comparable SNARC-like effect emerged in both experiments. Therefore, even in a context like that represented by stimuli consistently moving from right to left, which could potentially trigger a reversed right-to-left representation of visual speed, the canonical left-to-right representation persists. This finding aligns with the hypothesis that SNARC-like effects reflect stable associations between small magnitudes and left space, and large magnitudes and right space.

Despite the general similarity between the results of Experiments 1 and 3, some specific differences in RTs, contingent on speed and response side, are noteworthy. In Experiment 1, a statistically significant difference between RTs with the left-side and the right-side key was observed for the fast speed but not for the slow speed. Conversely, in Experiment 3, this pattern was reversed. This discrepancy might be related to another difference between the experiments: In Experiment 1, RTs were significantly shorter for the right-side key than for the left-side key, whereas in Experiment 3, the opposite was observed, although the difference did not reach statistical significance. A possible interpretation of this pattern of results will be provided in the General Discussion.

## Experiment 4

To further explore the generalizability of the left-to-right representation of visual speed, Experiment 4 combines features of the previous experiments. Similar to Experiment 2, the dots did not move in a consistent direction. However, their motion was restricted to the horizontal axis as in Experiments 1 and 3. Specifically, on each trial, all dots could move randomly, either from left to right or from right to left. Under the hypothesis that the spatial representation of visual speed is driven by the motion direction of the target stimuli, these opposing directions could result in the absence of a clearly defined spatial representation of visual speed (i.e., the absence of a SNARC-like effect). Conversely, a standard SNARC-like effect may still emerge under the hypothesis of a stable left-to-right representation of visual speed.

### Method

#### Participants

The criteria for the determination of the sample size were the same as in the previous experiments. Thirty students at the University of Padova took part in this experiment in exchange of course credits (*M*_age_ = 23.17 years, *SE* = 1.22, eight men). None of them had participated in the previous experiments. The participants had an average EHI score of 91.98 (*SE* = 2.54; range: 60–100). The EHI scores were consistent with self-declared handedness.

This study was approved by the Ethics Committee for Psychological Research at the University of Padova (approval number: 4480) and conducted following the ethical standards of the 1964 Helsinki Declaration. All participants read and signed a written informed consent.

#### Apparatus

The apparatus was the same as in Experiment 1.

#### Procedure

The procedure was the same as in Experiment 1, except that, on each trial, the motion direction of the dots in the RDKs could be either left-to-right or right-to-left. Motion direction was selected randomly, and all the dots consistently moved in the same randomly chosen direction. The direction of the RDKs representing the reference and target speeds could differ. However, the motion direction remained the same across the two successive presentations of the reference speed.

### Results and discussion

The data were analysed as in Experiment 1. Wrong responses (17.86% of trials) were removed and analysed separately, and no statistically significant effects emerged.^6^ All the participants performed above the chance level, with a maximum percentage of individual errors of 40.4%. Missed responses (0.68% of trials) were removed and not analysed further due to their low percentage. Outlier responses, defined as correct trials with RTs three standard deviations above or below the participant’s mean, were also removed (1.12% of trials).

**Fig. 5:**
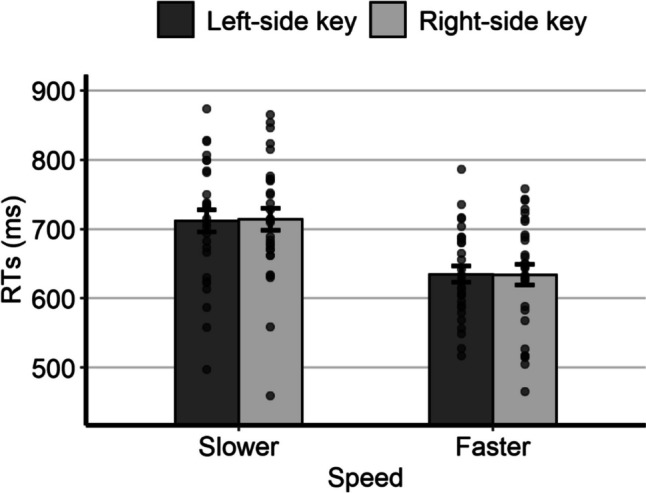
Mean individual RTs of Experiment 4 as a function of relative speed and response side. Error bars are *SEM*

The mean individual RTs as a function of relative speed and response side are represented in Fig. 5. As for the analysis of the possible SNARC-like effect, the model with the best fit to the RTs had, as fixed effects, the response side, the relative speed, and the interaction; as random effects it had the by-subject intercept, the by-subject slope for response side, the by-subject slope for the relative speed, and the by-item intercept. The main effect of relative speed was statistically significant, *F*(1,25.1) = 23.4, *p* < 0.001, due to shorter RTs for the fast speed (*M* = 637 ms, *SE* = 15.9) than for the slow speed (*M* = 717 ms, *SE* = 17.3). The main effect of response side was not statistically significant, *F*(1,25.7) = 0.08, *p* = 0.780, as nearly equal RTs emerged with the left key (*M* = 676 ms, *SE* = 13.9) and with the right key (*M* = 678 ms, *SE* = 15.6). Importantly, the interaction was not statistically significant, *F*(1,5712.3) = 0.71, *p* = 0.401, indicating the lack of a SNARC-like effect. For completeness, we report the mean RTs which, for the slow speed, were slightly shorter with the left key (*M* = 714 ms, *SE* = 16.8) than with the right key (*M* = 720 ms, *SE* = 18.6), whereas for the fast speed they were nearly identical with the right key (*M* = 637 ms, *SE* = 16.8) and with the left key (*M* = 638 ms, *SE* = 15.7).

As regards the distance effect, the model with the best fit with the data had the absolute difference between the speed of the target and the speed of the reference as a fixed effect, the by-subject intercept and slope as the random effects. The statistically significant negative regression coefficient confirms the existence of a distance effect, *b* = − 0.039, *SE* = 0.005, *t*(29.09) = − 8.50, *p* < 0.001.

We also directly compared the results of Experiment 4 and Experiment 1. The model had, as fixed effects, the experiment, the response side, the relative speed, and all the interactions; as random effects it had the by-subject intercept, the by-subject slope for response side, the by-subject slope for the relative speed, and the by-item intercept. We focus solely on the main and interaction effects of the experiment factor. There was a statistically significant main effect of experiment, *F*(1,57.9) = 7.10, *p* = 0.010, due to shorter RTs in Experiment 1 (*M* = 629 ms, *SE* = 14.5) than in Experiment 4 (*M* = 677 ms, *SE* = 14.5). There was also a statistically significant two-way interaction between experiment and relative speed, *F*(1,57.9) = 4.54, *p* = 0.037. In both experiments the RTs were shorter for the fast than for the slow speed, however the difference was more pronounced in Experiment 4 (*M* = − 79.7 ms, *SE* = 15.3, *p* < 0.001) than in Experiment 1 (*M* = − 58.0 ms, *SE* = 15.3, *p* = 0.001). The interaction between experiment and response side was not statistically significant, *F*(1,54.4) = 2.64, *p* = 0.110. Importantly, the three-way interaction was statistically significant, *F*(1,11,603.7) = 5.13, *p* = 0.024, reflecting the presence of a SNARC-like effect in Experiment 1 but not in Experiment 4.

Overall, the results of this fourth experiment support the hypothesis that the conflict between left-to-right and right-to-left motion can nullify the SNARC-like effect, indicating definite limits to the generalizability of the left-to-right representation of visual speed. Interestingly, a left-to-right representation emerged when the stimuli moved in entirely random directions (Experiment 2) and when they all moved in an opposite right-to-left direction (Experiment 3), but not when there was a conflict between left-to-right and right-to-left directions. A possible explanation for this finding will be provided in the General Discussion.

## General discussion

Previous studies have suggested that various dimensions, including size, luminance, time, and weight are mapped onto space, with smaller magnitudes represented on the left side of space and larger magnitudes on the right (see, e.g., Macnamara et al., 2018). The analogy between the spatial representation of numbers and nonnumerical magnitudes has inspired theories suggesting that processing numbers and nonnumerical magnitudes shares common underlying neural structures (a theory of magnitude’; Walsh, 2003, 2015). The present work explores a novel area within this framework: the possible spatial representation of visual speed. We believe that visual speed constitutes an interesting area of inquiry because, unlike other magnitudes, it is inherently related to space and spatial directions. This relationship allows for studying whether and how spatial-directional information provided by external cues can interact with the internal representation of magnitude along the horizontal axis.

In four experiments, participants were presented with RDKs showing a target speed and were asked to compare that speed with a previously seen reference speed, using lateralised response keys. All the dots in each RDK moved in a consistent direction (i.e., 100% motion coherence). However, the experiments differed regarding the motion direction of the dots. Specifically, the dots could move from left to right (Experiment 1), randomly across a 360° spectrum (Experiment 2), from right to left (Experiment 3), or randomly either from left to right or from right to left (Experiment 4). The results are summarised and discussed, beginning with the most theoretically relevant findings from the perspective of SNARC-like effects.

The first and most important result is the presence of convincing evidence supporting the hypothesis that a nonnumerical magnitude like speed, which is intrinsically connected with spatial directions, can also be spatially coded, giving rise to a SNARC-like effect. As evidenced by the results of Experiments 2, a left-to-right representation of visual speed can emerge not only when the motion direction of the stimuli aligns with this representation direction as in Experiment 1, but also when the spatial direction of the stimuli is entirely random, supporting the generalisability of the representation itself. What is even more interesting is that the left-to-right representation also emerged in Experiment 3, characterised by consistent right-to-left motion of the stimuli. According to the idea that spatial representations of numerical and nonnumerical magnitudes flexibly rely on the activation of working memory and adapt to the characteristics of the stimuli and the task, it could be predicted that a salient and consistent visual cue like the right-to-left direction of the moving dots could shape the direction of the spatial representation of visual speed. This would be consistent with previous studies showing that the SNARC effect can be modulated by the spatial and the serial position of numbers within a given framework (Bächtold et al., 1998; Fischer et al., 2010; Ginsburg & Gevers, 2015; Lindemann et al., 2008; Mingolo et al., 2021; van Dijck & Fias, 2011). However, the results of Experiment 3 indicate that visual speeds were consistently represented from left to right even when the stimuli moved in the opposite direction, supporting the stability and generalizability of the left-to-right representation.

At odds with the framework outlined so far, the lack of a SNARC-like effect in Experiment 4, in which the dots could move randomly either from left to right or from right to left, calls into question the generalizability and robustness of the left-to-right representation of visual speed. The results of Experiment 4 are reminiscent of observations by Wang et al. (2022) in their study on the SNARC-like effect for luminance. In their Experiment 4, they found no SNARC-like effect when participants judged the luminance of arrows randomly oriented to the left or right. They put forward a general hypothesis that spatial-directional information provided by visual stimuli in general could interfere with the construction of a spatial representation of the target magnitude, preventing the emergence of a SNARC-like effect. However, considering the results of our first three experiments, this hypothesis seems too general, as in those experiments a clear SNARC-like effect for visual speed emerged despite the clear directional information conveyed by the stimuli. This suggests that the interference of spatial-directional information with the spatial representation of nonnumerical magnitudes may not be as straightforward as previously thought and might be modulated by subtler perceptual-cognitive processes.

Our speculative hypothesis is that in our Experiment 4, as in Experiment 4 by Wang et al. (2022), participants perceived the stimuli as two distinct sets due to the opposing left-to-right and right-to-left directions, with each direction appearing as a separate category of motion. This categorization likely disrupted the formation of a continuous left-to-right spatial representation of speed, as participants may have unintentionally grouped the stimuli based on their orientation. In contrast, the fully random 360° directions in Experiment 2 did not encourage such a division, allowing for a more unified spatial representation. This difference suggests that when stimuli are limited to two opposing directions along a given axis (e.g., horizontal), participants may perceive them as two distinct groups rather than a single continuum. This interruption likely interferes with the SNARC-like mapping, which may require a unified representation of stimuli to emerge. This hypothesis highlights how the spatial representation of speed can be flexible and sensitive to specific task cues, and it suggests that the perception of stimuli as belonging to one single set might be essential for SNARC-like effects to appear. Further investigation could help clarify the necessary conditions for these effects and refine our understanding of how directional cues shape spatial magnitude representation.

A second important result is the emergence of a clear distance effect across all four experiments, evidenced by decreased RTs with increasing differences between the target speed and the reference speed. The distance effect is a general phenomenon in magnitude comparison, indicating that it is easier to compare quantities when they are further apart than when they are closer together (e.g., Moyer & Landauer, 1967). This effect suggests that participants effectively processed both the reference and target speeds in a consistent manner across all four experiments, as reflected by the narrow range of regression coefficients quantifying the distance effect, from − 0.035 (Experiment 1) to − 0.040 (Experiment 3). The robust presence of the distance effect in all experiments implies that any between-experiment differences in the spatial representation of visual speed cannot be attributed to differences in the basic process of magnitude comparison.

Third, in Experiments 1, 2, and 3, an interaction between relative speed and response side emerged, with the interaction pattern differing somewhat across the experiments. We will first compare the results of Experiment 1 (dots moving left-to-right) and Experiment 3 (dots moving right-to-left). In both experiments, a statistically significant two-way interaction emerged, indicating a SNARC-like effect, consistent with the hypothesis of a left-to-right representation of visual speed. However, in Experiment 1, a significant difference between left- and right-side responses emerged for fast speed but not for slow speed, whereas the opposite pattern was observed in Experiment 3. Additionally, in Experiment 1, responses were significantly faster with the right key than with the left key, while the opposite tended to be true in Experiment 3, although the difference was not statistically significant. Considering the characteristics of the stimuli in these experiments, we hypothesise that the differing results may reflect a Simon-like effect for motion direction. This phenomenon, described by Kerzel et al. (2001), suggests shorter RTs when the motion direction of a stimulus is compatible with the response side (e.g., a right key response for dots moving left-to-right) compared with when motion direction and response side are incompatible (e.g., a right key response for dots moving right-to-left). In the context of studies on SNARC-like effects, a Simon-like effect also emerged in the study by Wang et al. (2022), who used as stimuli static left and right arrows rather than moving objects.

In line with the Simon-like effect, in Experiment 1, the advantage for right-side responses would have emerged because the dots moved consistently from left to right. This advantage would have amplified the consequences of the SNARC-like effect for the fast speed. Specifically, the RTs for responses with the right key became even smaller than those with the left key, relative to the difference due to the SNARC-like effect. Concurrently, this advantage mitigated the consequences of the SNARC-like effect for the slow speed, decreasing the differences between RTs for the right and left keys, compared with the SNARC-like effect alone. The opposite pattern emerged in Experiment 3, as the dots moved from right to left, leading to an advantage for left-side responses.

The Simon-like effect is expected to equally influence the difference between RTs for left- and right-side responses for both slow and fast speeds. Thus, it would affect the main effect of response side and the results of post hoc tests, but not the magnitude of the interaction. This means the Simon-like and SNARC-like effects can be independently assessed. Notably, despite the lack of a SNARC-like effect in Experiment 4, an additional analysis revealed the presence of a Simon-like effect.^7^ For the sake of clarity, it is important to note that the results of Experiment 2 cannot be explained by a Simon-like effect. In this experiment, the dots did not move consistently along the horizontal axis, eliminating any compatibility or incompatibility between motion direction and response side. Despite this, similar to Experiment 1, a slight advantage for responses with the right-side key was observed, and a statistically significant difference in RTs emerged for fast speed but not for slow speed. A speculative explanation for these results involves a general effect of handedness: since the majority of participants were right-handed, a slight advantage for right-side responses was expected. This effect likely influenced the results of the other experiments to some degree as well.^8^

Finally, a consistent finding across the four experiments was that RTs were inversely related to target speed: Shorter RTs were observed for faster speeds, and longer RTs for slower speeds. This result aligns with previous studies that reported an inverse relationship between the speed of visual stimuli and RTs in classification tasks (Tynan & Sekuler, 1982) as well as in motor tasks (e.g., hitting a target moving at variable speeds; Smeets & Brenner, 1995; van Donkelaar et al., 1992). In the context of our experiments, it can be hypothesised that participants responded after the dots had travelled a minimum distance, which was reached earlier by fast-moving dots than by slow-moving dots. As this result is unrelated to the spatial representation of visual speed, we will not discuss it further.^9^

## Limitations and future directions

In all four experiments presented here, visual speed was treated as a task-relevant dimension, meaning that participants were explicitly asked to judge whether the target speed was slower or faster than a reference speed. We did not explore the possible emergence of a SNARC-like effect when visual speed was treated as an implicit dimension—that is, by focusing participants on a feature of the stimuli different from visual speed, manipulated independently of it. This would have been interesting because, according to the literature on the SNARC effect, the effect emerges not only when participants explicitly compare target and reference numbers (magnitude comparison task) but also when they judge whether a target number is even or odd (parity judgment task; see, e.g., Dehaene et al., 1993). This demonstrates that number magnitude is spatially coded even when it is irrelevant to the task. Regarding SNARC-like effects, a few studies have demonstrated that they can emerge even when the target magnitude is irrelevant to the task (e.g., Fumarola et al., 2014; Sellaro et al., 2015; Topić et al., 2022). However, the majority of studies have failed to reveal SNARC-like effects when the target magnitude was treated as an implicit dimension (e.g., Dalmaso et al., 2023b; Mariconda et al., 2022; Prpic et al., 2020; Vicovaro & Dalmaso, 2021; Wang et al., 2022).

Whether and under what conditions a spatial representation of nonnumerical magnitudes may emerge when implicit tasks are used remains an interesting open question. A relevant methodological problem is that, while the implicit task of parity judgment with numbers still requires participants to process the target magnitude at a relatively deep conceptual level, it is challenging to replicate the same level of conceptual processing in implicit tasks for nonnumerical magnitudes. For instance, an experiment could be designed where participants classify the colour of the dots rather than their speed and test if speed still affects the reaction times with left- and right-key responses. However, one problem with this approach is that participants would not need to process visual speed to complete the task. Therefore, a possible lack of a SNARC-like effect could be attributed to the nonautomatic nature of the spatial representation of speed or simply to the fact that observers overlooked visual speed in the task. Given that interpreting results from such implicit tasks remains challenging, we believe that suspending hypotheses concerning the automatic spatial representation of visual speed is prudent until an effective and convincing implicit task is found.

To sum up, future endeavours may focus on exploring the hypothesis that the spatial representation of nonnumerical magnitudes may critically depend on whether the stimuli are represented as belonging to different sets or a single set. Additionally, research should investigate the possibility that a spatial representation of visual speed may also emerge when visual speed is treated as an implicit dimension.

## Data Availability

Raw data are available on OSF (10.17605/OSF.IO/WNM9S). Hypotheses and methods were preregistered (https://aspredicted.org/W13_NJ6).

## Footnotes

1 SNARC-like effects have also emerged in tasks involving the classification of qualitative (i.e., nonphysical) dimensions, like face emotions (Holmes & Lourenco, 2011; Root et al., 2006), face ethnicity (Dalmaso et al., 2022) and economic value (Giuliani et al., 2021, 2024). However, it remains unclear whether spatial representations of these valence-related dimensions and the spatial representations of physical dimensions share the same underlying mechanisms. For instance, space–valence associations appear to be strongly modulated by participants’ handedness, with positive valence typically associated with the right side of space in right-handers and with the left side of space in left-handers (Casasanto, 2009, 2011; de la Vega et al., 2012; Kong, 2013). No evidence of such a strong modulation effect of handedness has so far emerged for SNARC-like effects involving numerical or physical non-numerical dimensions.

2 We have restructured the order of the experiments to ensure a smoother presentation flow.

3 Errors were analysed using generalized linear mixed effects models (R package *lme4*; Bates et al., 2015). The fixed effects were response side (left or right key), the relative speed of the target (slower or faster than the reference), and the interaction. The models differed only in their random components. The model with the best fit to the data had, as random effects, the by-subject intercept, the by-subject slope for speed, and the by-item intercept. No statistically significant main or interaction effects were found (*p* values > .110).

4 Errors were analysed as in Experiment 1 (see Footnote 3). The model with the best fit to the data had, as random effects, the by-subject intercept and the by-item intercept. No statistically significant main or interaction effects were found (*p* values > .188).

5 Errors were analysed as in Experiment 1 (see Footnote 3). The model with the best fit to the data had, as random effects, the by-subject intercept, the by-subject slope for the response side, the by-subject slope for speed, and the by-item intercept. Neither the main effect of response side [*z* = − 0.386, *p* = .700], nor that of relative speed [*z* = − 0.971, *p* = .331], were statistically significant. However, the interaction was statistically significant [*z* = − 2.157, *p* = .031], thus indicating the presence of a SNARC-like effect. For the slow speed, participants made significantly more errors when responding with the right key (*M* = 23.3%, *SE* = 1.74) than with the left key (*M* = 18.5%, *SE* = 1.50; *p* = .020). For the fast speed, the errors percentage was similar for the right key (*M* = 12.4%, *SE* = 1.26) and the left key (*M* = 11.7%, *SE* = 1.50; *p* = .700).

6 Errors were analysed as in Experiment 1 (see Footnote 3). The model with the best fit to the data had, as random effects, the by-subject intercept and the by-item intercept. No statistically significant main or interaction effects were found (*p* values > .222).

7 A linear mixed-effects model on RTs with compatibility as a fixed effect and random intercept for participants and items, showed that the RTs for compatible trials (left-to-right motion and right-side response key, or right-to-left motion and left-side response key; *M* = 662 ms, *SE* = 17.2) were significantly shorter than the RTs for incompatible trials (*M* = 685 ms, *SE* = 17.0); *F*(5761.6) = 22.83, *p* < .001.

8 This pattern also recalls what has been observed in auditory tempo perception, where a right-side advantage emerged for fast tempos, while slower tempos showed no such difference (De Tommaso & Prpic, 2020; Mariconda et al., 2024a; see also Wood et al., 2021). The consistency of this pattern can also be interpreted as an absence of spatial associations in the ‘slow’ range.

9 As suggested by one reviewer, another possible explanation is that the speed of the stimuli created a form of ‘pressure’ on participants, subtly encouraging them to ‘adapt’ to the presented speed. This adaptive response may have led participants to align their overall response latencies with the perceived pace, resulting in faster responses for high-speed stimuli as they unconsciously adjusted to the urgency of the faster movement.

## Code availability

Code will be made available upon request to the corresponding author.
